# Comparative Analysis of the Regulatory T Cells Dynamics in Peripheral Blood in Human and Porcine Polytrauma

**DOI:** 10.3389/fimmu.2018.00435

**Published:** 2018-03-13

**Authors:** Rafael Serve, Ramona Sturm, Lukas Schimunek, Philipp Störmann, David Heftrig, Michel P. J. Teuben, Elsie Oppermann, Klemens Horst, Roman Pfeifer, Tim P. Simon, Yannik Kalbas, Hans-Christoph Pape, Frank Hildebrand, Ingo Marzi, Borna Relja

**Affiliations:** ^1^Department of Trauma, Hand and Reconstructive Surgery, University Hospital Frankfurt, Goethe University, Frankfurt am Main, Germany; ^2^Department of Orthopaedic Trauma Surgery, University Hospital Zurich, University of Zurich, Zurich, Switzerland; ^3^Department of Abdominal and Visceral Surgery, University Hospital Frankfurt, Goethe University, Frankfurt am Main, Germany; ^4^Department of Orthopaedic Trauma, RWTH Aachen University, Aachen, Germany; ^5^Department of Intensive Care and Intermediate Care, RWTH Aachen University, Aachen, Germany

**Keywords:** regulatory T cell, lymphocyte, polytrauma, pig, porcine

## Abstract

**Background:**

Severely injured patients experience substantial immunological stress in the aftermath of traumatic insult, which often results in systemic immune dysregulation. Regulatory T cells (Treg) play a key role in the suppression of the immune response and in the maintenance of immunological homeostasis. Little is known about their presence and dynamics in blood after trauma, and nothing is known about Treg in the porcine polytrauma model. Here, we assessed different subsets of Treg in trauma patients (TP) and compared those to either healthy volunteers (HV) or data from porcine polytrauma.

**Methods:**

Peripheral blood was withdrawn from 20 TP with injury severity score (ISS) ≥16 at the admittance to the emergency department (ED), and subsequently on day 1 and at day 3. Ten HV were included as controls (ctrl). The porcine polytrauma model consisted of a femur fracture, liver laceration, lung contusion, and hemorrhagic shock resulting in an ISS of 27. After polytrauma, the animals underwent resuscitation and surgical fracture fixation. Blood samples were withdrawn before and immediately after trauma, 24 and 72 h later. Different subsets of Treg, CD4^+^CD25^+^, CD4^+^CD25^+^FoxP3^+^, CD4^+^CD25^+^CD127^−^, and CD4^+^CD25^+^CD127^−^FoxP3^+^ were characterized by flow cytometry.

**Results:**

Absolute cell counts of leukocytes were significantly increasing after trauma, and again decreasing in the follow-up in human and porcine samples. The proportion of human Treg in the peripheral blood of TP admitted to the ED was lower when compared to HV. Their numbers did not recover until 72 h after trauma. Comparable data were found for all subsets. The situation in the porcine trauma model was comparable with the clinical data. In porcine peripheral blood before trauma, we could identify Treg with the typical immunophenotype (CD4^+^CD25^+^CD127^−^), which were virtually absent immediately after trauma. Similar to the human situation, most of these cells expressed FoxP3, as assessed by intracellular FACS stain.

**Conclusion:**

Despite minor percental differences in the recovery of Treg populations after trauma, our findings show a comparable decrease of Treg early after polytrauma, and strengthen the immunological significance of the porcine polytrauma model. Furthermore, the Treg subpopulation CD4^+^CD25^+^CD127^−^ was characterized in porcine samples.

## Short Summary

Experimental trauma models represent a translational tool for the assessment of post-traumatic management. To verify the applicability of such models, we investigated the dynamics of regulatory T cells (Treg) in an experimental porcine severe multiple trauma (polytrauma) model and compared the findings with clinical data obtained from severely traumatized patients admitted to the emergency department of our institution. Different immunophenotypes of Treg, CD4^+^CD25^+^, CD4^+^CD25^+^CD127^−^, and CD4^+^CD25^+^FoxP3^+^ were characterized by flow cytometry before and immediately after trauma, 24 and 72 h later. The proportion of human Treg in trauma patients (TP) was lower compared to healthy volunteers, without showing significant recovery until 72 h after trauma. Data from the porcine trauma model confirmed the findings obtained in the clinic. In conclusion, Treg kinetics in peripheral blood in the porcine polytrauma model were comparable with the clinical situation in TP.

## Introduction

Trauma causes more than five million deaths annually and is described as the sixth leading cause of death globally and as number one cause of death in individuals under 40 years (1–3). About half of the patients die within the first 24 h after admittance to the emergency department (ED) due to severe traumatic injuries [injury severity score (ISS) ≥16, polytrauma], massive blood loss, serious organ damage, and/or craniocerebral injuries (4, 5). After this early phase, the leading causes of mortality are multiple organ dysfunction syndrome (MODS) and failure (MOF) (6–8). Organ failure is associated with a systemic immune reaction to tissue injury often termed as systemic inflammatory response syndrome (SIRS) (8). Trauma-associated immune response exceeds repair mechanisms and constricts the immunological defense at the site of trauma (9, 10). Hence, organ systems, which were not primarily injured are often affected by a compromised endothelial barrier, disturbed microcirculation, interstitial edema, tissue infiltration with leukocytes, and cell death, which results in the so-called remote organ injury, dysfunction, and/or failure (5, 6). SIRS is paralleled by a compensatory anti-inflammatory response syndrome (CARS), which is characterized by immunosuppression (5, 11). While patients may survive the early post-trauma phase, deaths caused by immunosuppression, e.g., nosocomial infections in the later clinical course are common (11, 12). However, the SIRS-CARS paradigm is rather a simplified concept of post-traumatic inflammation.

Regulating cellular and molecular mechanisms of the post-traumatic immunosuppression are widely unknown. Next to specific release of anti-inflammatory cytokines, such as IL-10, a shift toward a T-helper 2 lymphocyte-mediated response and/or lymphocyte anergy, regulatory T cells (Treg) contribute to the (post-traumatic) immunosuppression, and thus are strongly suggested to play a protruding role in its course (13–15). Treg play an important role in peripheral immune tolerance, and are able to alter innate and adaptive immune responses due to their ability to modulate and kill target cells, such as antigen-presenting cells and effector T cells, and to influence inflammatory cytokine environments and metabolic pathways (16, 17). So far, no consensus has been reached on the use of a specific set of markers when identifying Treg in human and mice. Since 1995, Treg were mainly characterized by their expression of CD4 antigen and IL-2 receptor α-chain, CD25 (18). Increased CD4^+^CD25^+^ Treg activity in severely injured patients has been found by MacConmara et al. (19) and applied to determine Treg expression profile. Since its discovery, the continuous expression of Treg-specific transcription factor forkhead box protein 3 (FoxP3) has been broadly used to characterize them as well (16, 20, 21). In trauma research, this set of markers has been used in experiments with human samples as well as in animal models (22, 23). Several other markers, such as CD127 (24), CTLA-4 (25), and GARP (26), have been proposed. Zhang et al. showed an increased proportion of CD4^+^CD25^+^CD127^low/−^ Treg regarding the overall number of lymphocytes over the course of 3 weeks in thoracic trauma patients (TP) (27). Yet, little is known about Treg dynamics after trauma regarding specific cell subsets. In the present study, expression of CD4, CD25, FoxP3, and/or CD127 were applied as specific markers for Treg to characterize their immunophenotype and dynamics after polytrauma.

Despite improvements in prognosis and survival, there is a lack of validated diagnostic tools for post-traumatic complications, e.g., sepsis (9). Moreover, a profound understanding of underlying immunological pathomechanisms in order to identify reliable diagnostic biomarkers and/or potential therapeutic targets is necessary (6, 9, 28, 29). Despite considerable advances in biomedical sciences and developing important but mostly murine disease and therapeutic models *in vivo*, there are concerning inconsistencies with regard to the interspecies analogy after polytrauma (30–32). These especially concern immunogenetics and immune functions, such as leukocyte subsets or cytokine pathways (33), which are criticized in murine models in the areas of humaninflamatory diseases, trauma, and various types of wound healing (31, 34). Therefore, porcine models (most commonly conducted with domestic pigs) are of increasing popularity because of their distinct physiological and anatomical comparability toward humans (34–36). In addition, the porcine immune system is probably best characterized after the ones of primates and mice (33). Hence, a porcine polytrauma model may contribute to better understanding of the pathophysiology of post-traumatic, e.g., SIRS and/or sepsis. Thus, in a comparative analysis in human and pigs, we have characterized the dynamics of the different Treg subtypes in peripheral blood after polytrauma.

## Materials and Methods

### Ethics

The patient study was performed in the University Hospital Frankfurt, Goethe-University, Germany, with the institutional ethical committee approval (312/10) in accordance with the Declaration of Helsinki and following STROBE-guidelines (37). The signed written informed consent form was obtained from all patients or their legally authorized representatives as well as from all included healthy volunteers. Twenty severely injured TP with an ISS ≥16 were enrolled. Exclusion criteria were age (younger than 18 or older than 80 years), acute myocardial stroke, cancer or chemotherapy, immunosuppressive drug therapy, HIV, infectious Hepatitis, acute CMV infection, and/or thromboembolic events. Upon admittance to the ED, vital signs were controlled and the ISS was calculated using the abbreviated injury scale (AIS) as of 2008 (38, 39). Ten healthy volunteers (HV) were included in the control group (ctrl). In total for the human study, the group size was *n* = 30.

All animal experiments were authorized by the responsible government authority (“Landesamt für Natur, Umwelt und Verbraucherschutz”: LANUV-NRW, Germany: AZ TV-Nr.: 84-02.04.2014.A265) and performed in compliance with the federal German law paying attention to the protection of animals. Animal experiments were performed at the Institute for Laboratory Animal Science & Experimental Surgery, RWTH Aachen University, Germany. Institutional Guidelines and the criteria in “Guide for the Care and Use of Laboratory Animals” (Eighth Edition The National Academies Press, 2011) were followed (40). All animals were handled in accordance with the ARRIVE guidelines (41). In total for the animal study, the group size was *n* = 10.

### Animals Experiments

Ten male German landrace pigs (*Sus scrofa*; 3 months old, 30 ± 5 kg) from a disease-free barrier breeding facility were included in this study and placed in air conditioned rooms for at least 7 days before the experiments. Prior experimentation, all animals were examined by a veterinarian, had free access to water, and were fasted overnight. For the benefit of the principles of the 3Rs (Replacement, Refinement, and Reduction), *in vivo* data presented in this manuscript were collected in the context of a larger study previously described in detail by Horst et al. (42).

Animals were pre-medicated with an intramuscular application of Azaperone (4 mg/kg, Stresni™, Janssen, Germany). Anesthesia was induced (3 mg/kg) and maintained with an intravenous injection of Propofol followed by orotracheal intubation (7.5 ch tube, Hi-Lo Lanz™) as described previously, and the animals were anesthetized during the whole observational period (42). Ventilated on volume-control mode (Draeger, Evita, Lübeck, Germany) with lung-protective ventilation as described before was applied (42). Catheters were aseptically inserted in the external jugular vein for fluids administration, anesthesia, and continuous monitoring of central venous pressure (CVP, central venous catheter 4-Lumen Catheter, 8.5 Fr., ArrowCatheter, Teleflex Medical, Germany), the right femoral vein to induce hemorrhagic shock (3-Lumen hemodialysis, 12.0 Fr., ArrowCatheter, Teleflex Medical, Germany) and into the femoral artery for blood pressure monitoring (4.0 Fr. arterial line catheter, Vygon, Germany). A urinary catheter in the bladder was used (12.0 Fr, Cystofix, Braun, Melsungen, Germany). For fluid management, crystalloid fluid (Sterofundin ISO^®^) were continuously applied (2 ml kg/BW/h). Prior to experimentation and after instrumentation and calibration all baseline measurements were performed.

Polytrauma was induced as described previously (42). Briefly, the animal was positioned on the right side and a femur fracture was induced with a bolt shot on the right hind leg (Blitz-Kerner, turbocut JOBB GmbH, Germany, 9 × 17, Dynamit Nobel AG, Troisdorf, Germany). Placed back in the dorsal position, the animals underwent blunt thoracic trauma with a bolt shot on the right dorsal lower thorax. Thereafter, a midline-laparotomy and a crosswise incision of the caudal liver lobe (4.5 cm × 4.5 cm) induced an uncontrolled bleeding for 30 s. The liver was packed using sterile gauze-compresses (10 cm× 10 cm). Hemorrhagic shock was induced by withdrawing blood from right the femoral artery until a mean arterial blood pressure of 40 ± 5 mm Hg was reached, and maintained for 90 min. During the shock phase, the animals reached a hypothermic state.

At the end of the polytrauma phase, resuscitation in accordance with established trauma guidelines (43, 44) by adjusting FiO_2_ to baseline values, and re-infusing the withdrawn blood and additional fluids (Sterofundin ISO^®^; 2 ml kg/BW/h) followed (45, 46). Rewarming using a forced-air warming system until normothermia (38.7–39.8°C) was applied (45). Clinical treatment of the open femur fracture was performed according to established trauma guidelines (44). Intensive care and complications management was applied according to the standardized clinical protocols from the latest recommendations of the European Resuscitation Council and Advanced Trauma Life Support (43). After the observational period for 72 h, the animals were euthanized by potassium chloride injection.

### Blood Sampling

Blood samples were withdrawn in prechilled ethylenediaminetetraacetic acid (EDTA) tubes (Sarstedt, Nürmbrecht, Germany) in pigs before trauma induction as control (ctrl), immediately after trauma induction (AT), 24 h (D1) and 72 h (D3) later. In TP blood samples were withdrawn in EDTA tubes directly after admission to the ED (AT) and 24 as well as 72 h later. The samples were kept on ice until their staining for flow cytometric analyses. Blood counts were obtained by standard clinical methods using the Sysmex XE-2100 automated blood cell counter (Sysmex Europe GmbH, Norderstedt, Germany) and are indicated as mean absolute cell count of leukocyte cells (ACL).

### Staining for Flow Cytometry

Human blood samples (100 µl) were transferred into polystyrene FACS tubes (BD Pharmingen™) and 5 µl of the mouse anti-human CD4 Brilliant Violett 421 (Clone RPA-T4), mouse anti-human CD25 APC-Cy7 (Clone BC96), and mouse anti-human CD127 PerCP-Cy5.5 (Clone HIL-7R-M21, BD, Franklin Lakes, NJ, USA) conjugated antibodies were added for 30 min at room temperature. Porcine blood samples were treated according to the same protocol, but other antibodies were used: mouse anti-pig CD4a Alexa Flour^®^ 647 (Clone 74-12-4, BD), mouse anti-pig CD25 (Mx405-conjugated) (Clone K231.3B2, Bio-Rad Antibodies, formerly known as AbD Serotec, Hercules, CA, USA), and polyclonal anti-IL7R FITC (CD127, antikoerper-online GmbH, Aachen, Germany). After the incubation, 2 ml phosphate buffered saline (PBS) was added and centrifugation at 400 × *g* for 5 min followed. Then, supernatants were removed and the samples were incubated 100 µl Fix & Perm Solution A (FIX&PERM Kit, An Der Grub) for 15 min at room temperature. Two milliliters of PBS were added and centrifugation at 400 × *g* for 5 min followed. Supernatants were removed and samples were incubated with 100 µl Fix & Perm Solution B and 5 µl of either monoclonal antibody FoxP3 PE-Cy7 (Clone PCH101) in human, or monoclonal antibody FoxP3 PE-Cyanine7 (Clone FJK-16s, Affymetrix eBiosciences, Santa Clara, CA, USA) in porcine samples for 30 min at room temperature. Subsequently, 2 ml of FACS lysing solution (FACS Lysing Solution, BD Pharmingen™) were added for additional 10 min. Centrifugation at 400 × *g* for 5 min, resuspension in 4 ml PBS supplemented with 0.5% bovine serum albumin (FACS buffer) and another centrifugation at 400 × *g* for 5 min followed. After removing the supernatants, cells were diluted in 500 µl FACS buffer and subjected to flow cytometric analyses using a BD FACS Canto 2™ and FACD DIVA™ software (BD). Lymphocytes were defined by gating in the corresponding forward and side scatter scan. The percentage of positive cells of the gated was determined.

### Statistical Analyses

The power analysis was conducted using an alpha of 0.05, a power of 0.80 for a two-tailed test. GraphPad Prism 6.0 software (GraphPad Software Inc., San Diego, CA, USA) was used to perform the statistical analysis. Data are given as mean and SEM in percentage of gated parent population. Normality distribution was assessed by D’Agostino–Pearson normality test. Then, Mann–Whitney *U* test or the matched-pair statistical analysis by using repeated measures ANOVA (Friedman test) with a Dunn’s *post hoc* test were applied to compare the differences between the groups. A *p*-value below 0.05 was considered statistically significant.

## Results

### Study Population

Twenty TP and 10 HV were enrolled. The majority of the study subjects were male (TP: 75% vs. HV: 70%). The mean age was 39.0 ± 3.5 in TP vs. 35.8 ± 4.0 in HV. Falls caused 40% of the mechanism of injury. All patients were substantially injured with a mean ISS of 30.5 ± 1.8. Within the patient cohort 40.0% had a head AIS ≥3 and 55.0% a chest AIS ≥3. Only one patient had an abdomen AIS ≥3 and 30.0% of patients had severe injuries of extremities. The mean stay in the intensive care unit was 10.8 ± 4.2 days. The in-hospital stay was 24.7 ± 5.7 days. Patients included in this study represent the cohort of major TP as shown in our previous studies (47, 48).

### Absolute Leukocyte Counts

**Figure 1:**
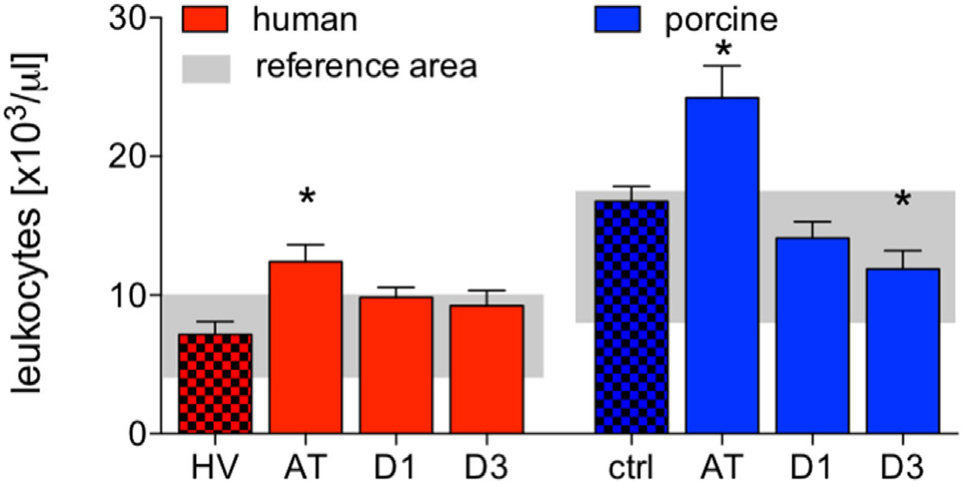
Analysis of absolute cell counts of leukocytes in human and porcine peripheral blood after polytrauma. Control samples were obtained from healthy volunteers (HV) or prior trauma (ctrl). Means and SEM are shown. AT: immediately after trauma; D1: 24 h after trauma; D3: 72 h after trauma; polytrauma patients: *n* = 20, HV: *n* = 10, animals: *n* = 10; *: significantly different (*p* < 0.05) to HV or ctrl, respectively.

Absolute cell counts of leukocytes were significantly increased after trauma compared to HV (12.4 ± 1.2 vs. 7.2 ± 0.9 cells ×10^3^/μl, *p* < 0.05). After 24 h and 3 days, leukocyte counts decreased to levels within the reference area (Figure 1).

Compared to controls, the absolute cell counts of porcine leukocytes significantly increased after trauma. Leukocytes decreased at D1 and D3, while the decrease at D3 was significant compared to ctrl (AT: 24.2 ± 2.3 and D3: 11.9 ± 1.3 vs. 16.7 ± 2.3 cells ×10^3^/μl, *p* < 0.05, Figure 1).

### CD4^+^ T Cells

**Figure 2:**
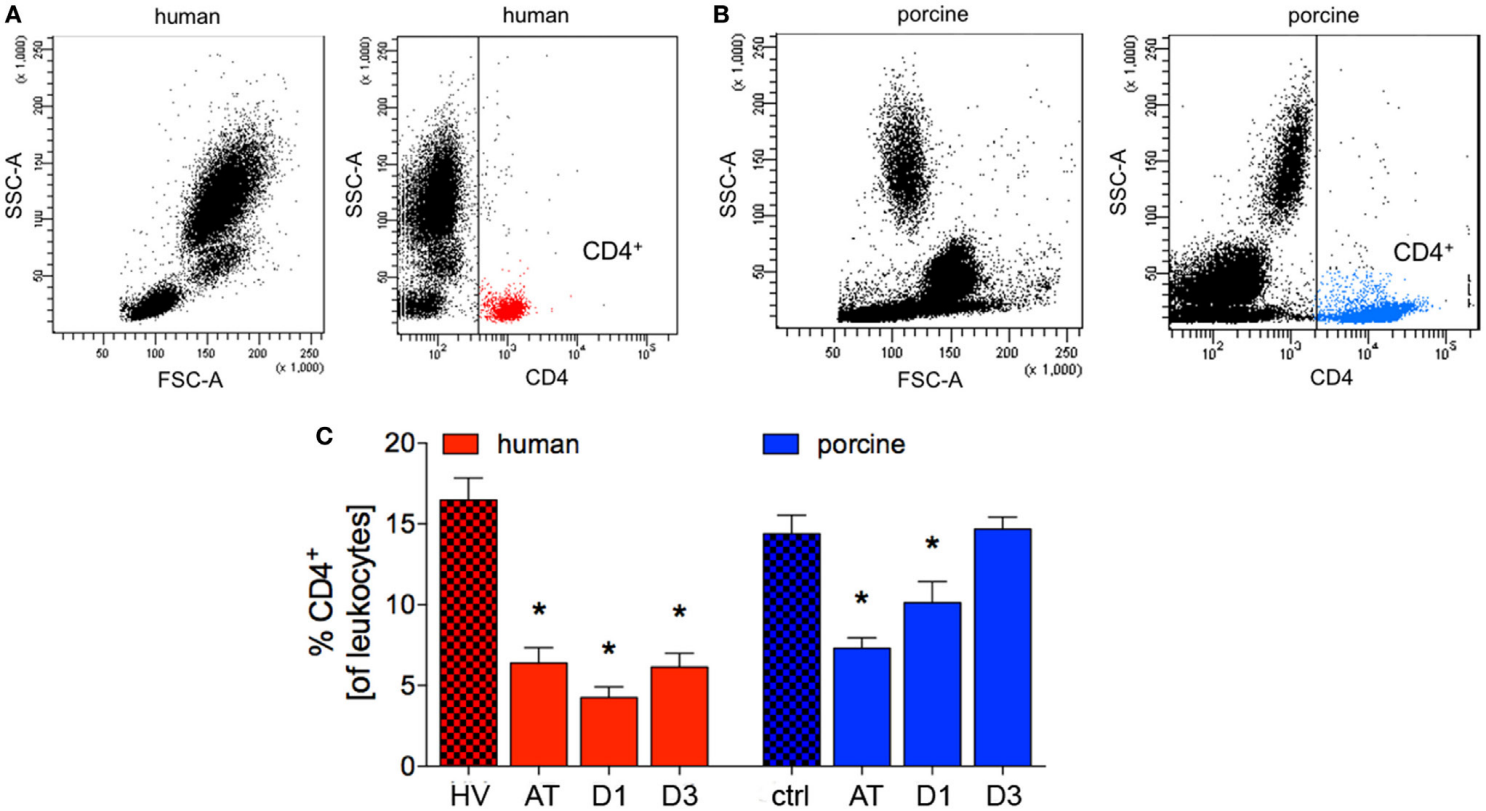
Analysis of CD4^+^ cells in human and porcine peripheral blood after polytrauma. **(A)** Dot plot analysis of peripheral blood of healthy volunteers (HV) stained with a human CD4 antibody. **(B)** Dot plot analysis of porcine peripheral blood obtained before trauma and stained with porcine CD4 antibody. **(C)** Relative counts of CD4 positive cells before and after trauma in percent. Means and SEM are shown. HV: healthy volunteers; AT: immediately after trauma; D1: 24 h after trauma; D3: 72 h after trauma; polytrauma patients: *n* = 20, HV: *n* = 10, animals: *n* = 10; *: significantly different (*p* < 0.05) to HV or ctrl, respectively.

The percentage of human CD4^+^ cells of all leukocytes was significantly reduced after trauma compared to HV (6.4 ± 0.9% vs. 16.5 ± 1.4%, *p* < 0.05; ACL: 1,181.1 vs. 792.5). A minimum value was reached in TP after 24 h (D1: 4.3 ± 0.6%, *p* < 0.05 vs. HV; ACL: 418.9 vs. 792.5). At the end of observation period, a significant difference in TP compared to HV was observed (6.1 ± 0.8% vs. HV, *p* < 0.05, Figures 2A,C; ACL: 567.6 vs. 792.5).

Compared to controls, the proportion of porcine CD4^+^ cells of all leukocytes showed a significant reduction after trauma and after 24 h (AT: 7.3 ± 0.6% and D1: 10.1 ± 1.3% vs. 14.4 ± 1.1%, *p* < 0.05; ACL: 2,413.4 vs. 1,773.4 and 1,430.4). At D3 after trauma no significant difference compared to ctrl were observed in pigs (Figures 2B,C). However, the mean absolute cell count remained at lower level at D3 (1,745.7).

### Total Lymphocytes

**Figure 3:**
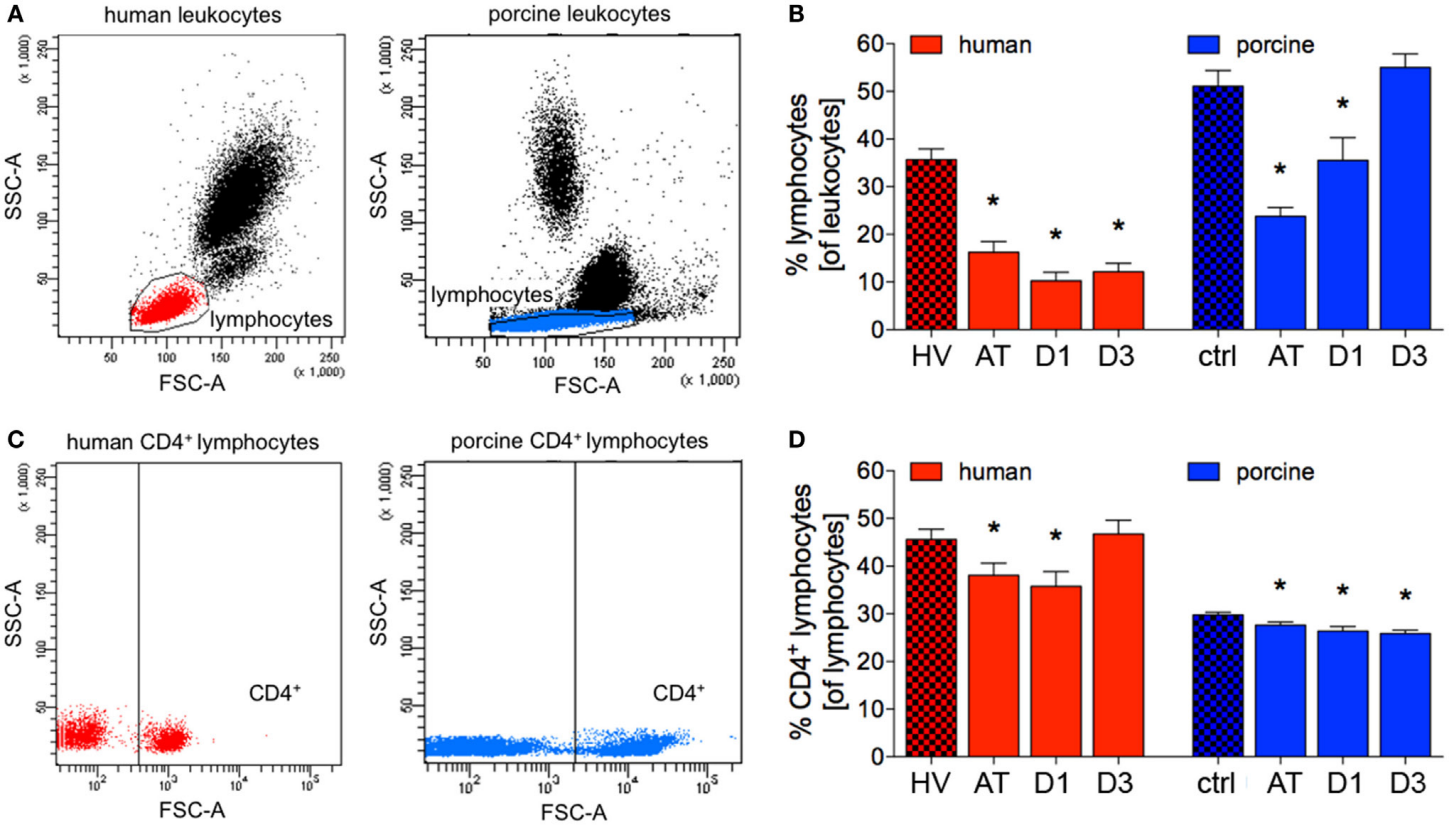
Relative counts of lymphocytes and CD4^+^ lymphocytes in human and porcine peripheral blood. **(A)** Gating strategy for lymphocytes before trauma. **(B)** Proportions of lymphocytes of all leukocytes before and after trauma. **(C)** Dot plot analysis of human and porcine peripheral blood obtained before trauma and stained with human and porcine CD4 antibody, respectively. **(D)** Relative count of CD4^+^ lymphocytes before and after trauma in percent. Means and SEM are shown. HV: healthy volunteers; AT: immediately after trauma; D1: 24 h after trauma; D3: 72 h after trauma; polytrauma patients: *n* = 20, HV: *n* = 10, animals: *n* = 10; *: significantly different (*p* < 0.05) to HV or ctrl, respectively.

The proportion of patients’ lymphocytes to all leukocytes significantly decreased after trauma compared to HV (16.3 ± 2.2 vs. 35.7 ± 2.3%, *p* < 0.05; ACL: 2,013.4 vs. 2,556.5), and a significant reduction remained at D1 and D3 in polytrauma patients compared to HV (10.2 ± 1.8 and 12.1 ± 1.8%, respectively, vs. 35.7 ± 2.3%, *p* < 0.05), Figures 3A,B; (ACL: 1,006.6 and 1,121.3 vs. 2,556.5).

The proportion of porcine lymphocytes of all leukocytes significantly decreased after trauma and at D1 compared to ctrl (23.8 ± 51.1 and 35.52 ± 4.8%, respectively, vs. 51.1 ± 3.3%, *p* < 0.05, Figures 3A,B; ACL: 5,766.7 and 5,015.4 vs. 8,562.7). At D3, the proportion of lymphocytes to leukocytes was comparable in polytraumatized pigs compared to ctrl (Figures 3A,B). However, the mean absolute cell count remained at lower level at D3 (6,545.0).

### CD4^+^ Lymphocytes

While the proportion of CD4^+^ lymphocytes of all lymphocytes was significantly reduced after trauma and at D1 in patients compared to HV (38.1 ± 2.6 and 35.8 ± 3.1%, respectively, vs. 45.6 ± 2.2%, *p* < 0.05; ACL: 766.5 and 360.2 vs. 1,165.5), no significant difference in percentages was observed at D3 (Figures 3C,D). However, the mean absolute cell count remained at lower level at D3 (523.6).

During the complete observational period of time, the proportion of CD4^+^ lymphocytes of all lymphocytes was significantly reduced in polytraumatized pigs compared to ctrl (AT: 27.6 ± 0.7%, D1: 26.4 ± 0.9%, and D3: 25.9 ± 0.7%, respectively, vs. 29.8 ± 0.5% in ctrl, *p* < 0.05, Figures 3C,D; ACL: 2,591.6, 1,324.1 and 1,693.8 vs. 2,551.6).

### CD4^+^CD25^+^ Regulatory to CD4^+^ T Cells-Ratio

**Figure 4:**
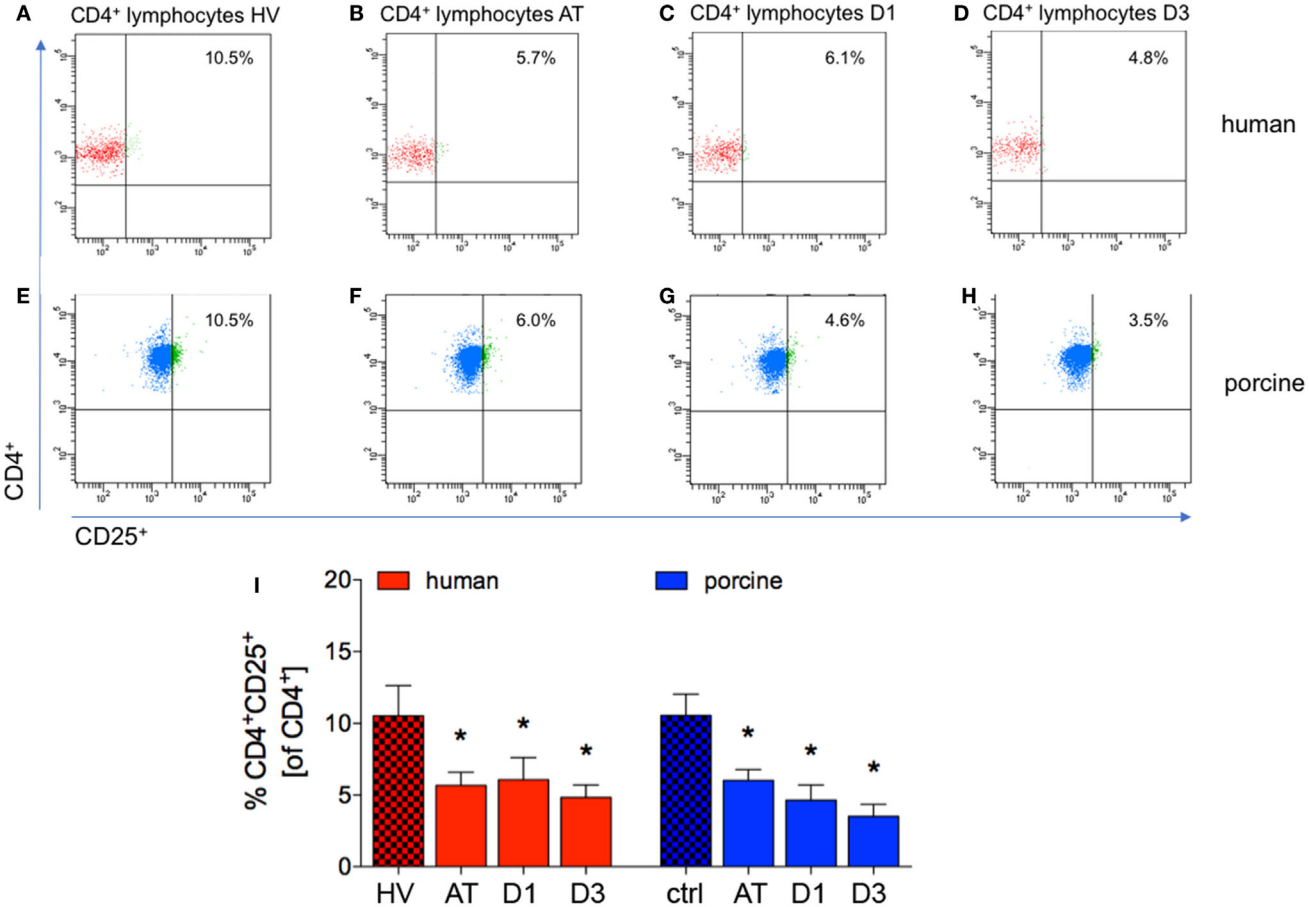
Proportions of CD4^+^CD25^+^ lymphocytes in peripheral blood before and after trauma. **(A–D)** Gating strategies for human CD4^+^CD25^+^ lymphocytes. **(E–H)** Gating strategies for porcine CD4^+^CD25^+^ lymphocytes. **(I)** Relative counts of CD4^+^CD25^+^ lymphocytes compared according to different time-points in percent. Means and SEM are shown. HV: healthy volunteers; AT: immediately after trauma; D1: 24 h after trauma; D3: 72 h after trauma; polytrauma patients: *n* = 20, HV: *n* = 10, animals: *n* = 10; *: significantly different (*p* < 0.05) to HV or ctrl, respectively.

The proportion of CD4^+^CD25^+^ to CD4^+^ cells significantly decreased directly after trauma and remained significantly lowered at D1 and D3 in patients compared with HV (AT: 5.7 ± 0.9%, D1: 6.1 ± 1.6%, and D3: 4.8 ± 0.9%, respectively, vs. 10.53 ± 2.1% in HV, *p* < 0.05, Figures 4A–D,I; ACL: 43.5, 21.9, and 25.3 vs. 122.7).

Immediately after trauma, and at D1 and D3 the proportion of CD4^+^CD25^+^ to CD4^+^ cells was significantly lower in pigs compared to ctrl (AT: 6.0 ± 0.7%, D1: 4.6 ± 1.1%, and D3: 3.5 ± 0.8%, respectively, vs. 10.55 ± 1.5% in ctrl, *p* < 0.05, Figures 4E–H,I; ACL: 95.8, 61.5, and 59.8 vs. 269.2).

### CD25^+^FoxP3^+^ Regulatory to CD4^+^ T Cells-Ratio

**Figure 5:**
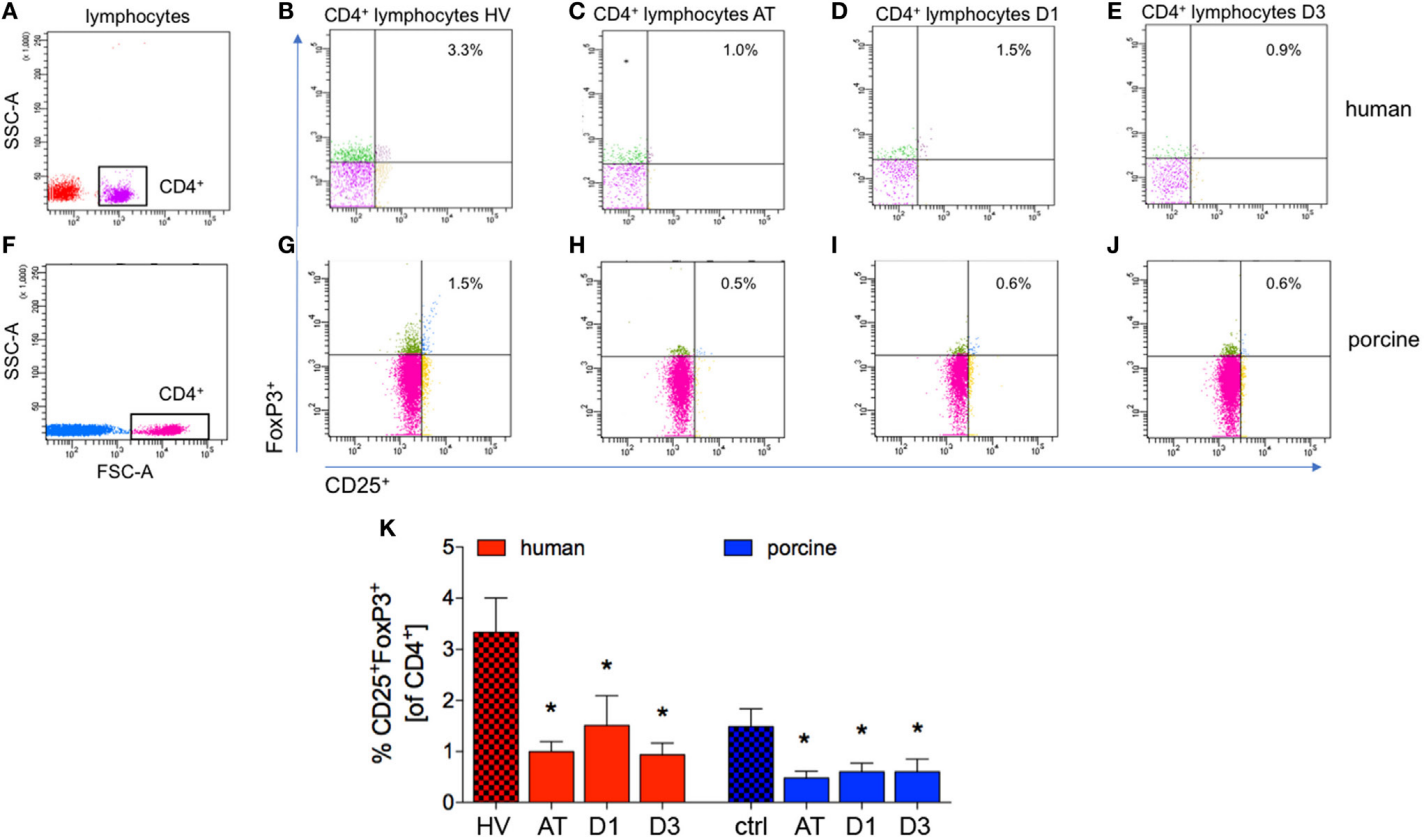
Analysis of CD25^+^FoxP3^+^ lymphocytes in CD4^+^ lymphocytes. **(A,F)** Gating strategy for CD4^+^ lymphocytes in human and porcine peripheral blood before trauma. **(B–E)** Gating strategies for human CD25^+^FoxP3^+^ lymphocytes. **(G–J)** Gating strategies for human CD25^+^FoxP3^+^ lymphocytes. **(K)** Relative counts of CD25^+^FoxP3^+^ lymphocytes in comparison in percent. Means and SEM are shown. HV: healthy volunteers; AT: immediately after trauma; D1: 24 h after trauma; D3: 72 h after trauma; polytrauma patients: *n* = 20, HV: *n* = 10, animals: *n* = 10; *: significantly different (*p* < 0.05) to HV or ctrl, respectively.

Compared to controls, the percentage of CD25^+^FoxP3^+^ cells of all CD4^+^ cells decreased significantly after trauma and during the complete observational period in patients compared with HV (AT: 1.0 ± 0.2%, D1: 1.5 ± 0.6%, and D3: 0.9 ± 0.2%, respectively, vs. 3.2 ± 0.7% in HV, *p* < 0.05, Figures 5A–E,K; ACL: 7.6, 5.4, and 4.9 vs. 38.9).

The proportion of CD4^+^CD25^+^FoxP3^+^ cells of all CD4^+^ cells was significantly lower at all analyzed time-points after trauma compared to ctrl in pigs (AT: 0.5 ± 0.1%, D1: 0.6 ± 0.2% and D3: 0.6 ± 0.3%, respectively, vs. 1.5 ± 0.4% in ctrl, *p* < 0.05, Figures 5F–J,K; ACL: 7.7, 7.9, and 10.2 vs. 37.9).

### CD25^+^CD127^−^ Regulatory to CD4^+^ T Cells-Ratio

**Figure 6:**
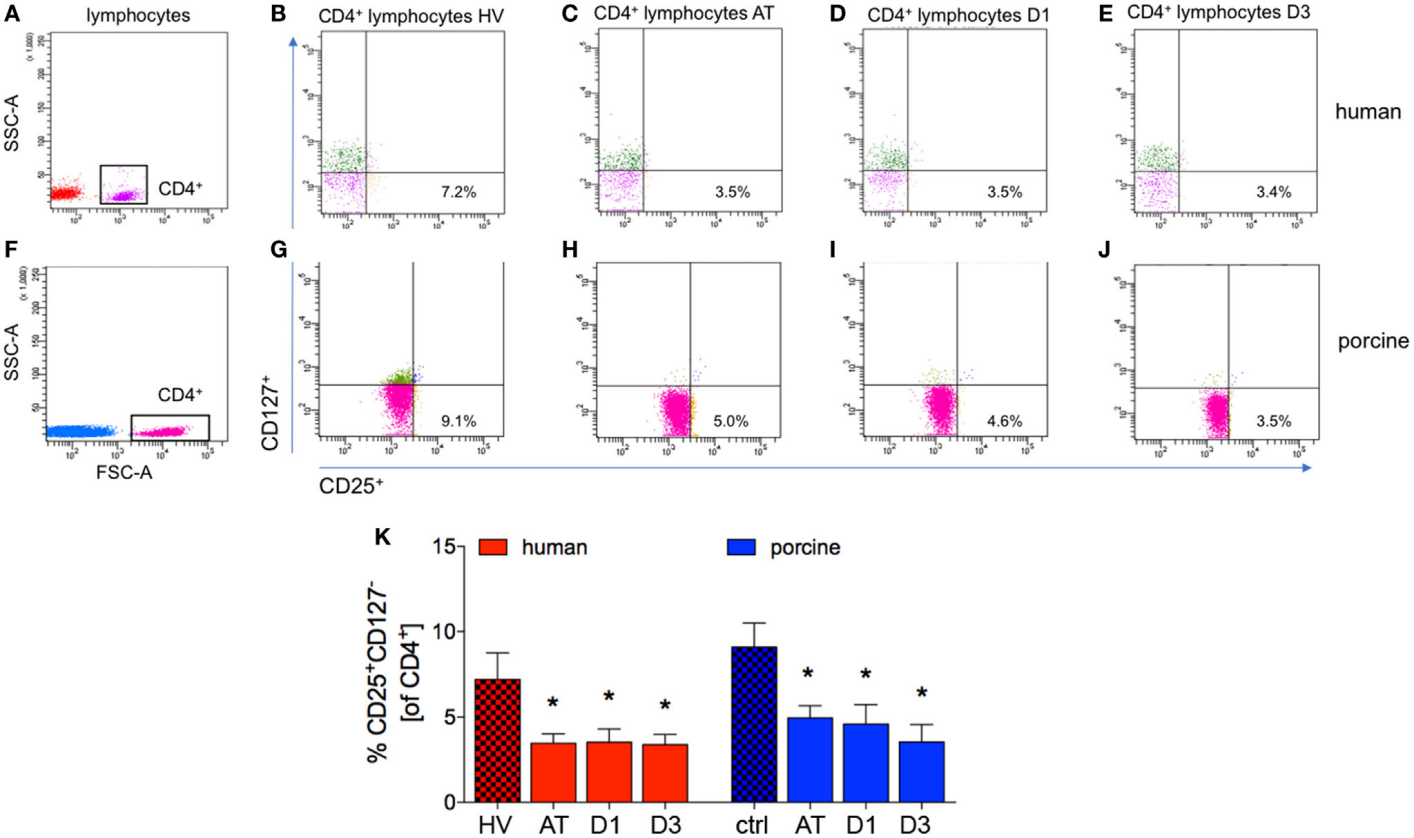
Analysis of CD25^+^CD127^−^ lymphocytes in CD4^+^ lymphocytes. **(A,F)** Gating strategy for CD4^+^ lymphocytes in human and porcine peripheral blood before trauma. **(B–E)** Gating strategies for human CD25^+^CD127^−^ lymphocytes. **(G–J)** Gating strategies for human CD25^+^CD127^−^ lymphocytes. **(K)** Relative counts of CD25^+^CD127^−^ lymphocytes in comparison in percent. Means and SEM are shown. HV: healthy volunteers; AT: immediately after trauma; D1: 24 h after trauma; D3: 72 h after trauma; polytrauma patients: *n* = 20, HV: *n* = 10, animals: *n* = 10; *significantly different (*p* < 0.05) to HV or ctrl, respectively.

After trauma, the proportion of CD25^+^CD127^−^ cells of all CD4^+^ cells dropped significantly at all analyzed time-points in patients compared with HV (AT: 3.5 ± 0.5%, D1: 3.5 ± 0.8% and D3: 3.4 ± 0.6%, respectively, vs. 7.2 ± 1.6% in HV, *p* < 0.05, Figures 6A–E,K; ACL: 26.6, 12.7, and 17.7 vs. 83.9).

The significant decrease of cells with a CD4^+^CD25^+^CD127^−^ subset in all CD4^+^ cells was observed in polytraumatized pigs during the whole observational period compared to ctrl (AT: 4.9 ± 0.7%, D1: 4.6 ± 1.1%, and D3: 3.5 ± 1.0%, respectively, vs. 9.1 ± 1.4% in ctrl, *p* < 0.05, Figures 6F–J,K; ACL: 78.8, 60.9, and 60.0 vs. 232.0).

### CD127^−^ Cells of CD4^+^CD25^+^FoxP3^+^ Regulatory Cells

**Figure 7:**
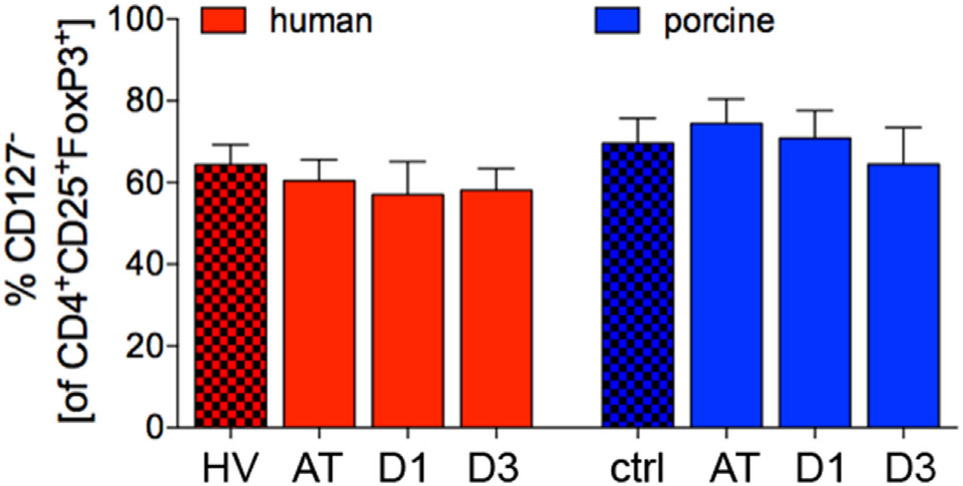
Proportion of CD127^−^ lymphocytes in CD4^+^CD25^+^FoxP3^+^ cells in human and porcine peripheral blood before and after trauma. Means and SEM are shown. HV: healthy volunteers; AT: immediately after trauma; D1: 24 h after trauma; D3: 72 h after trauma; polytrauma patients: *n* = 20, HV: *n* = 10, animals: *n* = 10.

No significant changes of the proportion of CD127^−^ cells of CD4^+^CD25^+^FoxP3^+^ appeared throughout the experiments in polytrauma patients compared to HV (AT: 60.4 ± 5.2%, D1: 57.0 ± 8.1%, and D3: 58.1 ± 5.3%, respectively, vs. 64.3 ± 5.0% in HV, *p* < 0.05, Figure 7).

The proportion of porcine CD127^−^ cells of CD4^+^CD25^+^FoxP3^+^ did not change significantly throughout the experiments com-pared to ctrl (AT: 74.5 ± 6.0%, D1: 70.8 ± 6.8% and D3: 64.5 ± 9.0%, respectively, vs. 69.6 ± 6.1% in ctrl, *p* < 0.05, Figure 7).

## Discussion

In the present study, we characterized a porcine Treg subtype CD4^+^CD25^+^CD127^−^ because a better differentiation of Treg from contaminating cell populations with similar expression patterns would allow for more precise and in-depth research. Furthermore, we compared blood kinetics of Treg between polytraumatized patients and a porcine model of polytrauma. It became clear that both the proportion of CD4^+^ cells and lymphocytes of all leukocytes were significantly lowered in peripheral blood samples after trauma. While this effect persisted in polytraumatized patients, a recovery in percentage, but not in absolute cell counts as comparable to patients, has been observed in pigs at 72 h after polytrauma. The proportion of CD4^+^ lymphocytes of all lymphocytes was significantly reduced immediately after trauma and after 24 h in TP, while such reduction was observed during the complete observational time period in pigs after polytrauma. Here again, absolute cell counts were reduced in both patients and pigs during the complete observational period. With regard to Treg, a significant reduction of each population CD4^+^CD25^+^, CD4^+^CD25^+^FoxP3^+^, or CD4^+^CD25^+^CD127^−^ cells was detectable after polytrauma in both patients and animals. Furthermore, no significant changes occurred in the proportion of CD127^−^ cells of CD4^+^CD25^+^FoxP3^+^ cells neither in humans nor in pigs. Therefore, we revealed that the early post-traumatic lymphocyte dynamics appear comparable between human and pigs after polytrauma. Furthermore, the various Treg subsets, which are commonly used for the Treg research, appear to have comparable immunophenotypes as well as dynamics between these two species.

Polytrauma induces a complex host immune response to tissue injury, a parallel pro- and anti-inflammatory state, bearing an elevated risk for infectious complications and/or MOF (5, 11, 49). Regulatory T cells play an important role in the orchestration of self-tolerance and immune reaction. Moreover, they have an important but not yet profoundly characterized role during the post-traumatic inflammation, and may, therefore, be an important determinant of the extent and/or severity of the post-traumatic immunosuppression as well (14, 50). Our understanding of basic immune mechanisms in hyperinflammatory states has much improved over the last decades, leading to a significant advancement in the clinical management during the first, predominantly hyperinflammatory phase (15). However, the immunosuppression is much less well understood, although it constitutes a severe clinical problem, since most patients dying in septic shock show signs of immunosuppression (51). Consequently, an important step in the treatment of infectious complications such as sepsis after trauma would imply an individualized therapy, considering interpersonal differences regarding long-term and acute immune status as well as the extent of trauma, the so-called trauma load or infection. We might be able to do so once we manage to obtain a detailed characterization of both immunosuppressive and immunostimulatory processes after trauma, as well as of specific clinical signs or immunological markers differentiating the patient’s state (11, 28, 51, 52). Therefore, we have characterized and analyzed the dynamics of currently known subsets of Treg, which may play an important role in the post-traumatic immunosuppression, and thereby the recovery from trauma.

In addition to detailed immune-surveillance data from patients, clinically relevant trauma models are required for this characterization. Also, any clinical intervention may have catastrophic consequences in the complex and critical post-traumatic situation. Therefore, preclinical evidence is essential. Murine models have been criticized for their inaccuracy in representing the human organism, especially regarding the immune system (31, 32, 53). Seok et al. systemically assessed the correlation of immune reactions caused by different insults (burn, trauma, and application of endotoxemia) to the human and the murine immune system (31). While in humans, they found a rather uniform pattern of immune reactions and changes of gene expression in immune effector cells, the patterns in mice were much less uniform and, more importantly, strongly different from the human situation, though there were some promising approaches (31, 54, 55). These demonstrated that gene expression patterns in mouse models closely recapitulated those in human inflammatory conditions and, moreover, strongly argued for the utility of mice as animal models of human disorders (54, 55). Summarized, a profound and specific characterization of models, and of species that are genetically more relevant due to their comparability to human inflammatory responses, is required. Porcine models not only offer practical clinical advantages over mice, but may also reflect the human situation much more adequately. The size of pigs provides large amounts of samples and allows for surgical procedures that are established in human medicine to be performed in experiments. Regarding anatomy, physiology, and genetics, pigs are more closely related to humans than rodents (32). The conservation of homology and structural motifs from pig, mice, and humans revealed that the similarity to human proteins was significantly higher for pigs (78%) compared to mouse (73%) (56). Data from numerous groups show the advantages of porcine models regarding wound healing (34), infectious diseases including sepsis (32), and immunological mechanisms involved in trauma and inflammation (53). Over the last few years, porcine models have, thus, especially gained the attention of researchers focusing on post-traumatic immunological stress (57–59). By strengthening the comparability of porcine and human immune systems, our findings may have implications for a range of further studies utilizing this model. In the literature, most experiments aimed to describe the post-traumatic immune function are conducted with patient-derived cells and rodent models, while only little work has been performed in other *in vivo* systems (23, 27). Most of these studies were performed with mice or rats (60–63). Thus, increased knowledge of the porcine model may help that specifically studies on the highly clinically relevant immunosuppression and inflammatory reactions after trauma will be performed in adequate model systems. Therefore, this study was initiated to better characterize Treg in pigs and to perform a comparability analysis with data obtained from patients after polytrauma.

A small number of studies investigated Treg in particular, but never in an experiment lasting longer than 4 h after trauma (22, 64). The porcine long-term polytrauma model with terminal analyses at 48 h after trauma has been described before, while Treg have never been addressed in a comparable trauma model before (65). In both, human as well as porcine polytrauma scenario, a general decrease in lymphocytes was observed in the present study. This effect may be explained by the post-traumatic cortisol release in order to confine the organism’s proinflammatory response to trauma. As a strong anti-inflammatory agent, it causes a discharge of neutrophils especially from the bone marrow and induces apoptosis in lymphocytes (66). In addition an increased extravasation of Treg into both lymphoid and non-lymphoid organs may explain these data. Therefore, further studies dealing with this issue are warranted. Regarding Treg specifically, a significant reduction of each analyzed subset was detected after trauma CD4^+^CD25^+^ (Figure 4), CD25^+^FoxP3^+^ (Figure 5), and CD25^+^CD127^−^ cells (Figure 6), and more importantly, it was visible in both patients and animals, strongly suggesting comparable Treg dynamics in both species after polytrauma. Our data are in line with the results obtained from a trauma model in rats conducted by Dai et al., consisting of a bilateral femur fracture and hemorrhagic shock (22). A significantly lower number of CD4^+^CD25^+^FoxP3^+^ Treg could be shown 4 h after trauma, when compared to ctrl and healthy controls (22). Experiments by other groups showed different results. MacConmara et al. reported no increase in the percentage of CD4^+^CD25^+^ Treg out of all CD4^+^ cells directly after trauma in humans, but a significant increase of their share on day 7 after trauma (19). Despite no changes in the percentage of Treg, the authors found a progressive increase in Treg suppressive activity regarding T cell proliferation in patients after severe trauma. Compared to Treg from healthy controls and patients 1 day after injury, CD4^+^CD25^high^ Treg from 7 days after injury had a significantly higher suppressive potency (19). In a mouse burn injury model, the suppressive potential of Treg from peripheral lymphoid tissues was assessed by examining the proliferation rate of lymph node cells (67). On day 1 after trauma, the Treg suppressive potential did not significantly differ in injured animals, while on day 7 after trauma, CD4^+^CD25^+^ Treg from injured mice exhibited a significantly increased activity compared to sham (67). In a porcine burn injury-induced sepsis model Zu et al. showed significantly decreased CD4^+^CD25^+^ Treg apoptosis rate in intestinal lymph nodes, demonstrating that the intestinal Treg may play an important role in the intestinal immune barrier system after severe burn injuries (68). In the last two studies, only the positive expression of CD4 and CD25 was used to characterize Treg, and due to this study design, it is not assured that specifically pure Treg were evaluated. In our study, we did not evaluate the immunosuppressive activity and functionality of Treg, and this remains to be elucidated in further studies. Gupta et al. found no significant change in the percentage of CD4^+^CD25^+^FoxP3^+^ Treg in blood samples collected instantly after admittance to ED from patients suffering from trauma (ISS = 18.71 ± 8.48) and hemorrhagic shock (23). However, we have included only severe polytrauma in both TP and pigs. Trauma load itself may have a significant influence on the Treg kinetics. Albertsmeier et al. found no significant change in the numbers of circulating CD4^+^CD25^+^CD127^−^ Treg two and 24 h after surgical trauma as compared to pre-operative cell counts (69). However, this study included a different trauma mechanism as well and, therefore, cannot reflect the polytrauma scenario. Analysis of peripheral blood withdrawn from patients with severe thoracic trauma revealed the percentage of CD4^+^CD25^+^CD127^−^ Treg in T-helper cells to be significantly higher than in healthy individuals from the first day until 3 weeks after trauma (27). In another study conducted on rats, Gore et al. compared the effects of lung contusion and the combination of lung contusion and hemorrhagic shock on peripheral CD4^+^CD25^+^FoxP3^+^ Treg concentration. Trauma caused no significant difference from control animals and furthermore no significant difference between the two trauma groups was observed (70). Summarized, these studies and our own data underline the importance of the adequate trauma model, and furthermore, they indicate that the trauma load as well as the trauma mechanism may play important roles for the Treg dynamics.

In addition, the comparable phenotyping of Treg is important, and due to differentially phenotyped Treg, the knowledge on this population is currently widely limited in the setting of polytrauma. Since the low expression of CD127 in CD4^+^CD25^+^ Treg was first described in 2006, it is increasingly used for their characterization (24, 71). Approximately 70% of porcine CD4^+^CD25^+^FoxP3^+^, and 65% of human CD4^+^CD25^+^FoxP3^+^ cells, respectively, were negative for CD127. In comparison, Seddiki et al. found 84% of CD4^+^CD25^+^ cells to be CD127^low^ in human peripheral blood (71). However, the authors did not stratify for the FoxP3 positive cells. Due to the fact, that no significant changes occurred in the proportion of CD127^−^ cells of CD4^+^CD25^+^FoxP3^+^ cells neither in humans nor in pigs after polytrauma, it appears reasonable, that these cells constitute the identical cell population.

Treg have not been a major focus of trauma research so far, but as they present themselves as a key player in many immune responses, they should be considered for more in-depth research in future. This could not only help to understand the etiology of the pathological post-traumatic immune response, but also, by the potential development of tools to influence the activity and/or functionality of Treg, new therapeutic models may be found to effectively alter immune responses at a profound level in general, as well as during the processes underlying, e.g., SIRS and sepsis (72, 73). Besides, markers to identify their state of action might shed light on the immunological status of polytraumatized patients. As we only assessed Treg in peripheral blood, the comparison to the situation in traumatized tissue might hold promising insights into their behavior after trauma. Cytokines that are important for Treg functionality (e.g., IL-2) or are secreted by them (e.g., IL-10, TGFβ, IL-35), respectively, could be investigated in both peripheral blood and traumatized tissue to further understand the interplay between Treg and other players of the immune response.

Limitations of study certainly include several methodical issues. Also due to the limited hardware settings, we did not include additional analyses, such as the live/dead cell discrimination assay or functional analyses. Furthermore, the intensities of the staining sometimes appear somewhat low, and though the instrumental settings were performed by using isotype controls as well as unstained samples, this fact limits the interpretations of certain cell populations. Another potentially interesting issue may be the gender-based difference in the Treg immunophenotyping. In this study 20 TP (15 males vs. 5 females) and 10 healthy volunteers (7 males vs. 3 females) have been included, and no potential gender-based differences were found. However, the power is not enough to verify any female-vs.-male differences. This certainly remains an interesting point for the future studies.

The porcine polytrauma model has been introduced in recent years, but due to its practical benefits, such as great amounts of samples and applicability of techniques used in human medicine, and the similarity of human and porcine immune systems, it has caused much attention and will surely play an important role in future. Our results strengthen the significance and reliability of the porcine model by characterizing similarities in immunophenotypes between porcine and human Treg after polytrauma.

## Treat Research Group

**B. Auner**, Department of Trauma, Hand and Reconstructive Surgery, University Hospital Frankfurt, Goethe University, Frankfurt am Main, Germany; **L. Egerer**, Department of Trauma Surgery, Technical University Munich, Germany; **M. V. Griensven**, Department of Trauma Surgery, Technical University Munich, Germany; **A. Haug**, Department of Trauma Surgery, Technical University Munich, Germany; **F. Hildebrand**, Department of Orthopaedic Trauma, RWTH Aachen University, Germany; **K. Horst**, Department of Orthopaedic Trauma, RWTH Aachen University, Germany; **M. Huber-Lang**, Institute of Clinical and Experimental Trauma-Immunology, University of Ulm, Germany; **Y. Kalbas**, Department of Orthopaedic Trauma Surgery, University Hospital Zurich, University of Zurich, Switzerland; **M. Kalbitz**, Department of Orthopedic Trauma, Hand-, Plastic-, and Reconstructive Surgery, University of Ulm, Germany; **G. Marx**, Department of Intensive Care and Intermediate Care, RWTH Aachen University, Germany; **I. Marzi**, Department of Trauma, Hand and Reconstructive Surgery, University Hospital Frankfurt, Goethe University, Frankfurt am Main, Germany; **H. C. Pape**, Department of Orthopaedic Trauma Surgery, University Hospital Zurich, University of Zurich, Switzerland; **R. Pfeifer**, Department of Orthopaedic Trauma Surgery, University Hospital Zurich, University of Zurich, Switzerland; **K. Reiss**, Institute of Pharmacology and Toxicology, RWTH Aachen University, Germany; **B. Relja**, Department of Trauma, Hand and Reconstructive Surgery, University Hospital Frankfurt, Goethe University, Frankfurt am Main, Germany; **T. P. Simon**, Department of Intensive Care and Intermediate Care, RWTH Aachen University, Germany; **P. Störmann**, Department of Trauma, Hand and Reconstructive Surgery, University Hospital Frankfurt, Goethe University, Frankfurt am Main, Germany; **M. Teuben**, Department of Orthopaedic Trauma Surgery, University Hospital Zurich, University of Zurich, Switzerland; **R. Tolba**, Institute for Laboratory Animal Science and Experimental Surgery, RWTH Aachen University, Germany; **S. Uhlig**, Institute of Pharmacology and Toxicology, RWTH Aachen University, Germany.

## Ethics Statement

All information have been provided in the manuscript.

## Author Contributions

BR designed the study, obtained the ethical approval for human analyses, established the methods, performed the statistical analysis, and revised the manuscript. RS, the first author collected samples, carried out analyses, and made the first draft of the manuscript. RS, the first author, LS and MT collected the samples from the animal experiments and carried out analyses. RS and DH collected the samples from the human experiments and carried out analyses. EO contributed significantly to the sample analyses. MT, KH, PS, RP, YK, TPS carried out animal experiments. HCP, FH, and IM contributed intellectually to the completion of the study.

## Conflict of Interest Statement

The authors declare that the research was conducted in the absence of any commercial or financial relationships that could be construed as a potential conflict of interest.
